# Single-spoke binning: Reducing motion artifacts in abdominal radial stack-of-stars imaging

**DOI:** 10.1002/mrm.29576

**Published:** 2023-01-03

**Authors:** Ivo T. Maatman, Sjoerd Ypma, Marc Kachelrieß, Yannick Berker, Erik van der Bijl, Kai Tobias Block, John J. Hermans, Marnix C. Maas, Tom W. J. Scheenen

**Affiliations:** 1 Department of Medical Imaging, Radboud University Medical Center, Nijmegen, The Netherlands; 2 Division of X-Ray Imaging and Computed Tomography, German Cancer Research Center (DKFZ), Heidelberg, Germany; 3 Hopp Children’s Cancer Center Heidelberg (KiTZ), Heidelberg, Germany; 4 Clinical Cooperation Unit Pediatric Oncology, German Cancer Research Center (DKFZ) and German Cancer Consortium (DKTK), Heidelberg, Germany; 5 National Center for Tumor Diseases (NCT) Heidelberg, Heidelberg, Germany; 6 Department of Radiation Oncology, Radboud University Medical Center, Nijmegen, The Netherlands; 7 Department of Radiology, NYU Langone Health, New York New York, USA

**Keywords:** FID navigators, motion binning, motion correction, radial sampling, respiratory gating

## Abstract

**Purpose:**

To increase the effectiveness of respiratory gating in radial stack-of-stars MRI, particularly when imaging at high spatial resolutions or with multiple echoes.

**Methods:**

Free induction decay (FID) navigators were integrated into a three-dimensional gradient echo radial stack-of-stars pulse sequence. These navigators provided a motion signal with a high temporal resolution, which allowed single-spoke binning (SSB): each spoke at each phase encode step was sorted individually to the corresponding motion state of the respiratory signal. SSB was compared with spoke-angle binning (SAB), in which all phase encode steps of one projection angle were sorted without the use of additional navigator data. To illustrate the benefit of SSB over SAB, images of a motion phantom and of six free-breathing volunteers were reconstructed after motion-gating using either method. Image sharpness was quantitatively compared using image gradient entropies.

**Results:**

The proposed method resulted in sharper images of the motion phantom and free-breathing volunteers. Differences in gradient entropy were statistically significant (p = 0.03) in favor of SSB. The increased accuracy of motion-gating led to a decrease of streaking artifacts in motion-gated four-dimensional reconstructions. To consistently estimate respiratory signals from the FID-navigator data, specific types of gradient spoiler waveforms were required.

**Conclusion:**

SSB allowed high-resolution motion-corrected MR imaging, even when acquiring multiple gradient echo signals or large acquisition matrices, without sacrificing accuracy of motion-gating. SSB thus relieves restrictions on the choice of pulse sequence parameters, enabling the use of motion-gated radial stack-of-stars MRI in a broader domain of clinical applications.

## 1 | INTRODUCTION

MRI of the abdomen is sensitive to artifacts from respiratory motion, which can obscure information vital for clinical decision-making.^1^ Two common ways to reduce these motion artifacts are to acquire MR data during breath holds or to trigger an MR acquisition on an (external) motion signal. Acquiring MRI data during breath hold restricts acquisition times to approximately 15–20 s, which limits attainable spatial resolution and volumetric coverage of routinely used fast three-dimensional (3D) gradient echo (GRE) sequences. These limitations can be alleviated by using multiple breath holds per image volume, at the cost of increased patient discomfort, decreased compliance, low scan efficiency, and the possibility of spatial mismatch between partial image sets. The challenges are exacerbated in sequences employing long repetition times, such as multi-GRE T*_2_-quantification of the liver in iron-overload disorders or T*_2_-weighted imaging of iron oxide-based contrast agents for the detection of lymph node metastases.^2–4^ Triggered acquisitions, in which data are collected only at specific intervals selected according to a respiratory signal, are sensitive to inaccuracies of the respiratory signal and patient motion between successive shots, and suffer from a low duty cycle. Therefore, there is a need for MRI methods which acquire data continuously during free breathing, while delivering high-resolution, motion-corrected and artifact-free images.

Non-Cartesian sequences employing, for example, spiral or radial readout schemes have been proposed to facilitate continuous free-breathing data acquisition, as these are intrinsically less sensitive to motion.^5,6^ One such sequence adopted in an increasing number of clinical applications is the radial stack-of-stars 3D GRE sequence. In this sequence, k-space is sampled along stacked partitions of intersecting radial readouts, resulting in cylindrical k-space coverage.^6^ This scheme greatly reduces the sensitivity to respiratory motion, but standard motion-averaged image reconstructions still suffer from motion blur. However, by using golden angle increments between subsequent stacks of radial readouts the data can be sorted into multiple phases (bins) according to a surrogate motion signal, while a roughly uniform distribution of projection angles across motion phases is maintained.^7^ The image reconstruction of the sorted data is often based on compressed sensing^8,9^ or low-rank tensor completion,^10,11^ exploiting the image sparsity in a transform domain or the high correlations across different image dimensions, respectively.

By explicitly accounting for the motion in the reconstruction process, static or motion-resolved images can be reconstructed with strongly reduced motion corruption, a method that has found its way to clinical applications such as liver fat or fibrosis imaging,^12^ contrast-enhanced imaging of the upper abdomen,^13,14^ and four-dimensional (4D) motion-resolved imaging for radiotherapy treatment planning.^15^ The motion surrogate may be obtained from external sensors or navigator signals, but is often derived directly from the k-space data using self-gating, in which motion information is extracted from low-frequency signal content acquired near the k-space center.^16^

Self-gating has been applied successfully to multiple k-space trajectories,^16–19^ but suffers from some limitations in the case of radial stack-of-stars. Data sorting for the radial stack-of-stars sequence is usually performed by assigning all spokes acquired at a specific angle to the same motion phase, a method we refer to as spoke-angle binning (SAB). Through this approach, image reconstruction can be performed separately per slice, which is computationally efficient. However, SAB-based image reconstructions display motion blur or aliasing if significant motion occurs during the time interval Δ*t* needed to acquire all readouts at a given spoke angle. This time interval is set by the repetition time and the number of partitions, the latter of which defines spatial coverage and resolution along the slice axis. The SAB approach therefore results in a trade-off between volumetric coverage, spatial resolution, and image contrast in motion-corrected radial stack-of-stars imaging.

In this work, we resolve this trade-off by binning k-space data into motion phases per individual spoke, a method we refer to as single-spoke binning (SSB). To acquire a motion signal with sufficient temporal resolution, we sample free induction decay (FID) navigators at every *T*_R_. This novel strategy allows for effective motion-gating of the MRI data, even for many partitions or long repetition times. We illustrate the possibilities of our new approach with a motion phantom experiment, and in vivo demonstrate a significant quantitative difference between self-gated SAB and FID-navigated SSB approaches in motion-gated inverse nonuniform Fourier transform (iNUFFT) reconstructions.

## 2 | METHODS

**FIGURE 1:**
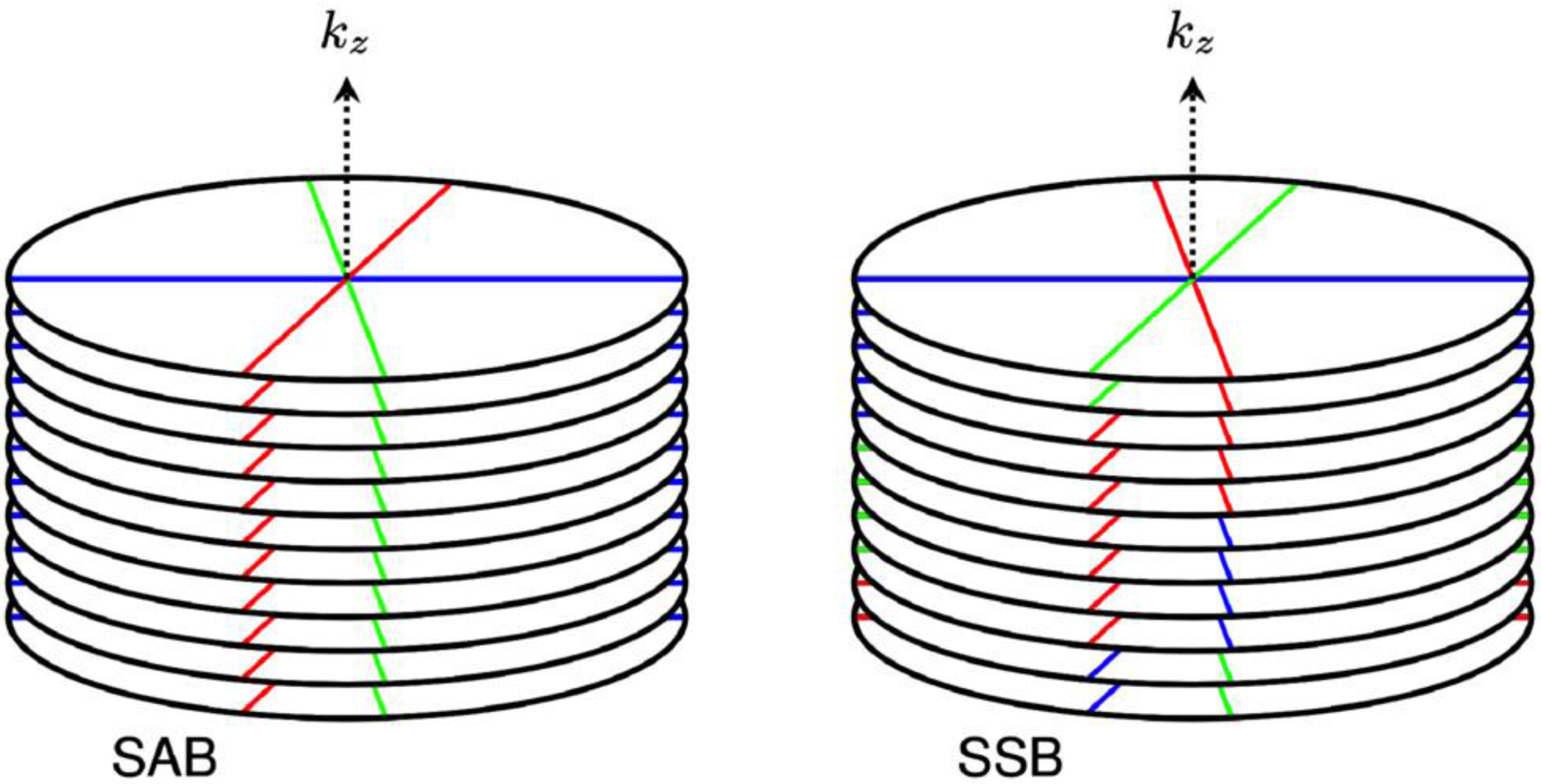
Schematic drawing of the two motion-binning strategies for a golden-angle radial stack-of-stars acquisition for three motion phases (indicated by colors) and three spoke angles. In spoke-angle binning (SAB), all readout lines corresponding to a spoke angle are assigned to the same motion phase. In single-spoke binning (SSB) each readout line is individually assigned to a motion phase. Thus, different readouts acquired at the same spoke angle may be assigned to different motion phases.

The new SSB method was compared to SAB by creating motion-gated images from a radial stack-of-stars pulse sequence using one of three methods: self-gated SAB, FID-navigated SAB, or SSB. With SAB, all phase-encoding steps per spoke angle were assigned to a single motion phase. Meanwhile, with SSB, binning was performed on a single-spoke level, allowing each phase-encoding step to be binned separately (Figure 1).

Four modifications were made to the pulse sequence in order to enable SSB: incorporation of an FID navigator, use of a randomized partition sampling order, use of a water-excitation pulse, and implementation of an alternative gradient-spoiling scheme (see Section 2.1 for details on all four modifications). Respiratory signals were obtained either from the FID navigator data or directly from the k-space data. All SSB reconstructions used only the respiratory signals obtained from the FID navigator data as inputs, while SAB reconstructions were performed using either the self-gated or FID-navigated respiratory signals. To obtain accurate and stable respiratory signals from the FID navigator data, the gradient-spoiling scheme was optimized using scans of a stationary phantom. The severity of motion artifacts was investigated for self-gated SAB, FID-navigated SAB, and SSB using measurements of a motion phantom. Using data obtained from six free breathing volunteers, image sharpness of self-gated SAB and FID-navigated SSB reconstructions were compared quantitatively with a gradient entropy criterion.

### 2.1 | Pulse sequence

**FIGURE 2:**
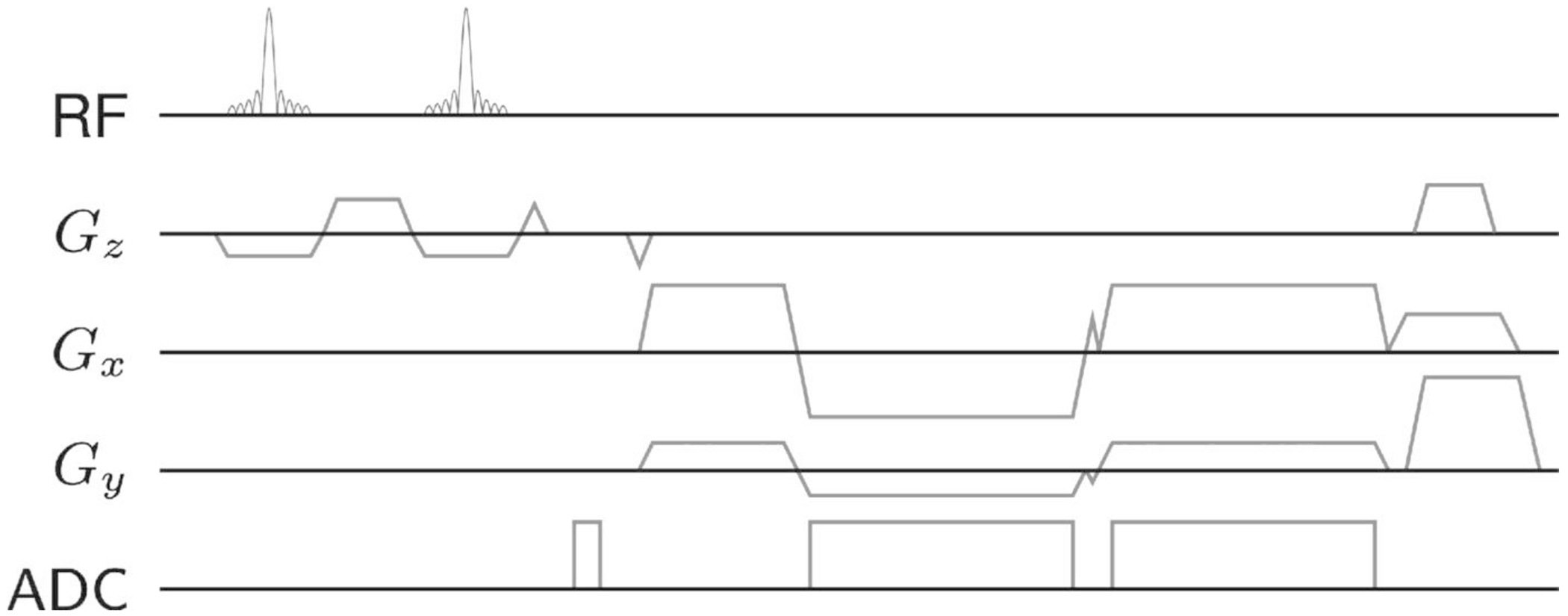
Diagram of the modified multi-echo golden-angle radial stack-of-stars spoiled gradient echo pulse sequence. Excitation is performed using a 1-1 binomial slab-selective water-excitation pulse. A short, nonlocalized free induction decay acquisition between slab-selection rephasing and slice encoding acts as respiratory navigator. Small triangles on axes *G*_*x*_ and *G*_*y*_ represent the blip gradients, which added slight rotations between subsequent gradient echo readouts. Spoiler gradients were timed with slight offsets to avoid exceeding gradient stimulation limits.

The 3D GRE pulse sequence utilized was a modified version of the golden-angle radial stack-of-stars sequence described in References 6,8 (Figure 2). An FID navigator acquisition consisting of 32 data points sampled in 200 μs was inserted between the excitation and phase-encoding gradients for each *T*_R_. All slice encoding steps were acquired for every spoke angle before switching to the next angle, applying a golden-angle rotation of 111.25° between subsequent spoke stacks. In case of multi-echo acquisitions, a bipolar readout scheme was used. Blip gradients were applied between echoes to offset multi-echo readouts by 2°, reducing the coherence of streaking artifacts in multi-echo reconstructions.^20^

**FIGURE 3:**
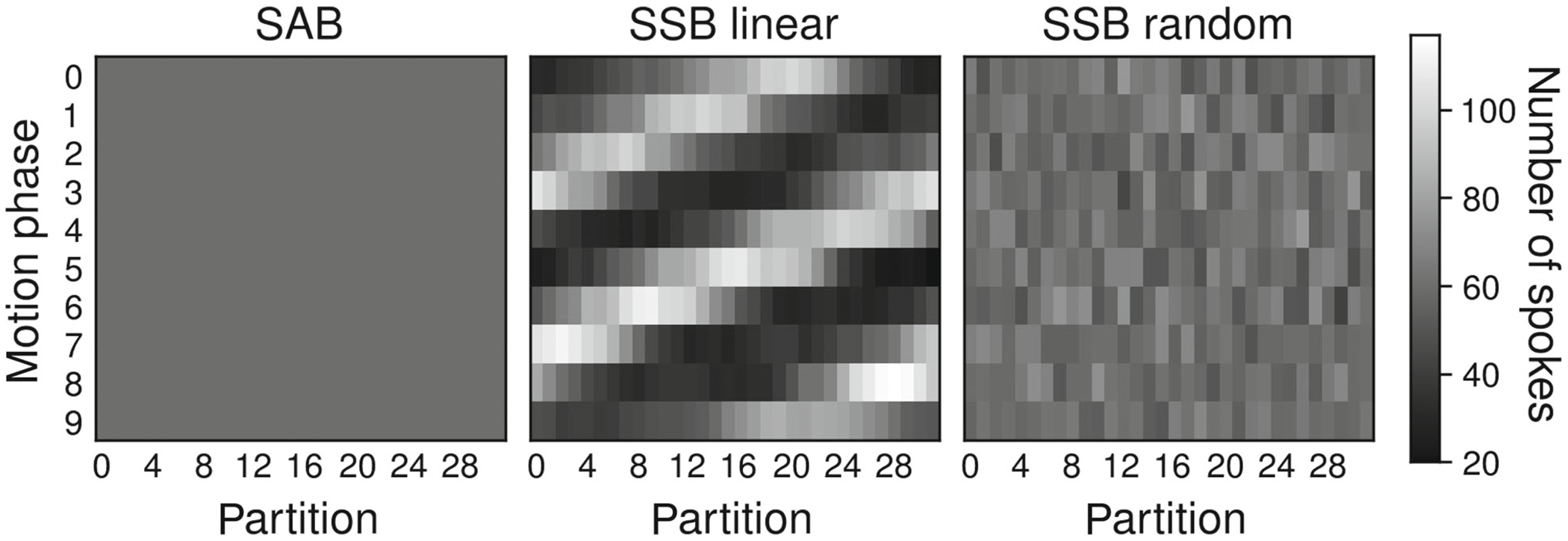
Simulated distributions of the number of spokes across 10 unique motion phases and 32 partitions for spoke-angle binning (left), single-spoke binning (SSB) with linear *k*_*z*_-reordering (center), and SSB with random *k*_*z*_-reordering (right). Linear *k*_*z*_-reordering results in strongly varying numbers of spokes between partitions in any given motion phase. This effect can be suppressed by sampling partitions in random order (right).

In SSB, readouts originating from a single spoke angle are assigned to different motion states to increase the accuracy of the motion gating. However, sampling partitions (*k_z_*) in a linear order introduces a nonuniformity in the distribution of readouts over partitions and motion phases, stipulated by the respiratory frequencies (Figure 3). Therefore, partitions were sampled in a random order at each spoke angle to ensure more evenly distributed readouts per partition and motion phase.

In general, radial MRI acquisitions require some form of fat suppression or a very high readout bandwidth to prevent blurring of off-resonant fat signals.^21^ However, a “quick” fat-saturated acquisition^22,23^ is difficult with a randomized partition order, as it leads to inconsistent levels of fat suppression between spoke angles within each partition. On the other hand, a high bandwidth would reduce the SNR of the images significantly. Thus, water excitation with a 1-1 pair binomial pulse was used to prevent fat blurring.^24^ RF spoiling was performed with a 50° phase-difference increment.^25^

Finally, the gradient spoilers were altered to enable robust detection of respiratory signals from the FID navigator data. In the standard sequence, an angle-dependent spoiling scheme is used that combines readout and spoiler gradients into a single gradient waveform.^6^ This angular dependence of the spoiler gradients results in a correlation between the FID navigator signal and the spoke angle, which can be seen as peaks in the power spectrum at frequencies corresponding to cycles of the angular variation.^17,21,26^ The positions of these spectral peaks depend on *T*_R_ and the number of partitions *N*_p_, as these parameters set the cycles’ duration, and thus lead to a parameter-dependent disturbance of the navigator signals. To mitigate this effect, two alternate spoiling schemes, using fixed and random spoiler gradient moments, were implemented and compared to angle-dependent spoiling using phantom measurements. Details of these measurements are described in Section 2.4.1.

### 2.2 | Respiratory signal detection

Two types of respiratory signals were obtained, either from the FID navigator data, or directly from the imaging data. First, respiratory signals with temporal resolutions of *T*_R_ were estimated from the navigator data obtained from each receiver coil. The first and last five sample points of every FID readout were discarded, as these showed system-dependent fluctuations with high amplitude. The magnitude values of the remaining 22 FID samples were averaged separately for each of the *N*_c_ receiver coils. Remaining eddy-current effects were eliminated by subtracting a signal baseline from the *N*_c_ time curves,^27^ and a low-pass filter was used to remove high-frequency components unrelated to respiratory motion. The respiratory signal was extracted as the first principal component, that is, by projecting the data onto the first unit basis vector of the set of coil signals.

Second, to evaluate the merit of the navigators, self-gated respiratory signals with temporal resolutions of *N*_p_*T*_R_ were extracted from the k-space data, that is, through self-gating. Therefore, to obtain representations of the low-frequency signal content, the magnitudes of the nine central samples of each readout were averaged and the remaining readout points were discarded.^28^ The *N_c_* time curves were corrected for eddy currents^27^ and filtered with a 3-point moving average. A principal component analysis was performed to extract the motion signals, in this case on a matrix $B\in {\mathbb{R}}^{M\times n}$. Here, *m* equals the number of spoke angles and *n* = *N*_p_*N*_c_*N*_e_, that is, the number of acquired partitions times the number of coils times the number of signal echoes. The first unit basis vectors were assumed to represent respiratory motion.

### 2.3 | Reconstruction

#### 2.3.1 | 3D iNUFFT reconstruction

Three-dimensional image volumes were reconstructed by resampling the acquired k-space onto a rectilinear grid and applying a 3D inverse Cartesian Fast Fourier Transform. A ramp filter was applied to the data for density compensation, and a two-dimensional Kaiser–Bessel kernel was used in the gridding process. A phase shift was applied to the k-space data to correct for gradient delays during the readouts. The shift amount was determined via a calibration prescan.^29^ The coil sensitivities were estimated from low-resolution 3D iNUFFT reconstructions of the motion-averaged data.^30^ Complex-valued coil images were combined through root sum-of-squares.

#### 2.3.2 | 4D iNUFFT reconstruction

The free-breathing data were binned into motion phases based on the amplitude of the respiratory signal to create 4D gated iNUFFT reconstructions. The boundaries of the signal’s end-exhalation bin were chosen to minimize the variation in this phase. The boundaries of the other bins were determined by requiring each bin to have an equal number of spokes.

Since the number of spokes in the SSB reconstructions varied per partition and motion phase, an additional density compensation was applied to the spokes before the gridding step. To this end, the signal in each partition was divided by the number of spokes it contained after motion binning. Each motion phase was then reconstructed with the 3D iNUFFT.

#### 2.3.3 | 4D iterative reconstruction

Because the k-space sample distribution per motion phase can differ greatly between SAB and SSB, both reconstructions lead to different aliasing patterns in the case of undersampling. Therefore, the effect of SSB on the performance of compressed sensing was investigated with iterative reconstructions.

In order to avoid any image blurring due to regularization across motion phases, compressed sensing reconstructions of motion-gated data were performed for each motion phase separately, using a total variation constraint over the three spatial dimensions. Specifically, the following equation was solved:^31^

(1) $$\underset{f}{\arg\min}∥\text{Ψ}(f(r)){∥}_{1}\text{subject to}∥XSf-p{∥}_{2}^{2}<\epsilon ,$$

where $f\in {\mathbb{R}}^{M\times 1}$ is the vectorized 3D image estimate for a particular motion phase, with *M* denoting the number of voxels times the number of receiver coils. The vector $p\in {\mathbb{C}}^{N\times 1}$ represents the *N* k-space data points assigned to this motion phase times the number of receiver coils.$X\in {\mathbb{C}}^{N\times M}$ is the forward NUFFT operator that maps the complex image data to the radial sampling pattern of the reconstructed motion phase. ϵ∈ℝ and $S\in {\mathbb{C}}^{M\times 1}$ are the raw data fidelity threshold and the coil sensitivities, respectively. The regularization term is the $\ell_{1}$-norm of the spatial gradient of the image volume:^28^

(2) $$\|\text{Ψ}(f(r)){\|}_{1}=\underset{x,y,z}{\sum }[\frac{1}{\text{Δ}x^{2}}{\left(f_{x,y,z}-f_{x-\text{Δ}x,y,z}\right)}^{2}{+\frac{1}{\text{Δ}y^{2}}{\left(f_{x,y,z}-f_{x,y-\text{Δ}y,z}\right)}^{2}+\frac{1}{\text{Δ}z^{2}}{\left(f_{x,y,z}-f_{x,y,z-\text{Δ}z}\right)}^{2}]}^{1/2},$$

where Δx, Δy, and Δz, represent the physical distances between adjacent voxels along the three spatial axes. Minimization of Equation (1) is described in detail in References 28,32.

### 2.4 | Measurements

MR data were acquired for a stationary phantom, a motion phantom, and six volunteers using the modified golden-angle radial stack-of-stars sequence (Table 1) on 3T systems (MAGNETOM Prisma Fit [*n* = 5] and MAGNETOM Skyra [*n* = 1], Siemens Healthineers). All measurements were performed using standard 32-channel spine and 18-channel body array coils, and all acquisitions were carried out in transverse orientation, that is, with *k_z_* parallel to the scanner central axis.

#### 2.4.1 | Stationary phantom

A stationary phantom was measured with three different gradient spoiling schemes in order to find a scheme with minimal systematic variations in FID navigator magnitudes. Candidate spoiling schemes used fixed, random,^33^ or angle-dependent^6^ spoiler-gradient moments. The maximum moments of all spoiler gradients were set to half of the readout moment. Fixed gradient moments were always of the set size and of equal polarity throughout the sequence run time. In the fixed and random cases, rephasing of the last readout moments was incorporated into the spoiler gradient. Random gradient moments were calculated by sampling the amplitudes and signs from a uniform distribution without replacement. With angle-dependent spoiling, readout rephasing was omitted to minimize *T*_R_.

The influence of the three spoiling schemes on the navigators were analyzed by plotting the FID signal curves and calculating their variances. For the calculation of one variance, the signal magnitudes for the 20 different receiver channels were normalized to unit-less quantities:

(3) $${\tilde{M}}_{c,s}(t)=\frac{M_{c,s}(t)}{\mu_{c,s}},$$

where $M_{c,S}(t)$ and $\mu_{c,s}$ are the magnitude and mean of the navigator data, respectively, for receiver *c* and spoiling scheme *s*. This normalization allowed the data of all coils to be used in the calculation of the three variances. The mean variances and SDs were then calculated across the set of receivers. Furthermore, MR data were reconstructed with 3D iNUFFTs to investigate the effect of the spoiling schemes on image quality.

#### 2.4.2 | Motion phantom

MR data of a motion phantom (Quasar MRI 4D, Modus QA) were acquired to investigate the accuracy of the motion detection and the binning process. The phantom contained a central oscillating gel-filled cylinder, programmed to describe a respiratory-like motion pattern with 15 cycles per minute and a maximum peak-to-peak translation of 3 cm. In the MR acquisitions, *T*_R_ and *N*_p_ were chosen such that self-gating was expected to produce inaccurate motion estimates. Magnetization was spoiled with fixed gradient moments along all spatial axes. Other acquisition parameters are summarized in Table 1.

Two respiratory signals were estimated from the MR data, that is, from the FID navigators and from the k-space data. Both signals were synchronized with the motion phantom’s reference by maximizing their cross-correlation functions. These maximum correlation coefficients were used as accuracy measures for the extracted respiratory signals.

The motion-phantom data were binned into 20 overlapping motion phases, where each motion phase contained 10% of the data, leading to an undersampling factor of approximately 10 in each. Binning was done in three ways. First, as a reference, SAB was performed using the k-space data, that is, using self-gating (“self-gated SAB”). Second, to assess the effect of the two motion binning methods independently of the respiratory signals, SAB was also performed based on the FID navigator data (“navigated SAB”). In this case, the motion bins were selected based on the average FID navigator’s signal across partitions. Third, SSB was performed using the FID navigators. All binned motion phantom data were then reconstructed using 4D iNUFFTs.

#### 2.4.3 | In vivo

In vivo evaluation of the proposed method was performed by acquiring multi-echo golden-angle radial stack-of-stars data in six free-breathing volunteers. This volunteer study was conducted with approval of the local Ethics board, and written informed consent was obtained from all volunteers. For each volunteer, the number of acquired partitions was adjusted to cover the liver and a large part of the lungs. This led to values of Δ*t* between 690 and 920 ms, which is generally too long for accurate respiratory motion binning using self-gated SAB. The magnetization was spoiled with fixed gradient moments along all three spatial axes.

To investigate the effects of SSB on the performance of compressed sensing, 4D image volumes of the first echoes were reconstructed iteratively. The 4D iterative reconstructions applied either self-gated SAB, FID-navigated SAB, or SSB. k-Space data were binned into 12 overlapping motion phases, where each motion phase contained 17% of the data, corresponding to an undersampling factor of approximately 3.5. This undersampling factor ensured that the aliasing artifacts could be removed with the iterative reconstruction or greatly reduced by summing iNUFFT images of multiple echoes.

### 2.5 | Quantitative evaluation of image sharpness

The image sharpness resulting from self-gated SAB and SSB was quantitatively compared. To create fully sampled images in every motion phase without image blur from spatial regularization, multi-echo images were combined by calculating the root sum-of-squares of 4D iNUFFTs along the echo axis. By doing so, relative intensities of the streaking artifacts were reduced, because the k-space trajectories of signal echoes were separated by the blip angle. This approach mixed the contrast of the different GREs, but it eliminated the need of regularization to reduce the aliasing.

The levels of motion-induced artifacts in these images were estimated with the gradient entropy *H*(*g*) as metric,^34^ represented by:

(4) $$H(g)=-\underset{i,j}{\sum }g_{ij}\log_{2}g_{ij},$$

where g∈ℝ is a normalized image gradient calculated symmetrically along the vertical image axis,^34^ and *i, j* run through all coordinates in *g*. Generally, this entropy criterion lowers as the number of dark pixels in the image gradients increases. Respiratory motion during the MR acquisition typically has an opposite effect on these gradient images, causing regions of decreased sharpness to show increased intensity.^35^

First, regions of interest (ROIs) were selected from the images of one volunteer in transversal, coronal, and sagittal orientations to illustrate the efficacy of the gradient entropy as a measure for motion blur. Each ROI had a size 50 × 50 pixels. The ROIs were chosen near veins in the lungs, at the liver dome, and the kidneys. The ROIs were selected on the basis of being clinically relevant while also expected to display some level of motion artifacts. *H*(*g*) was calculated for each of the ROIs and 12 motion phases. Second, gradient entropies were evaluated per motion phase for each of the six volunteers. In this case, *H*(*g*) represented the average gradient entropy across the 70 central coronal slices. For statistical analysis, values for the volumetric *H*(*g*) were averaged across the motion phases per volunteer. This produced six pairs of independent variables suited for statistical comparison with a nonparametric two-sided paired Wilcoxon signed-rank test. A p-value < 0.05 was considered to indicate a statistically significant difference.

### 2.6 | Implementation

Respiratory signals were extracted using a custom Python program, while the iNUFFT and iterative reconstructions were performed using C++.^28^ Reconstructions were carried out on a two-socket Intel Xeon Gold 6130 CPU with a total of 32 cores and 378 GB of memory.

## 3 | RESULTS

### 3.1 | Stationary phantom

**FIGURE 4:**
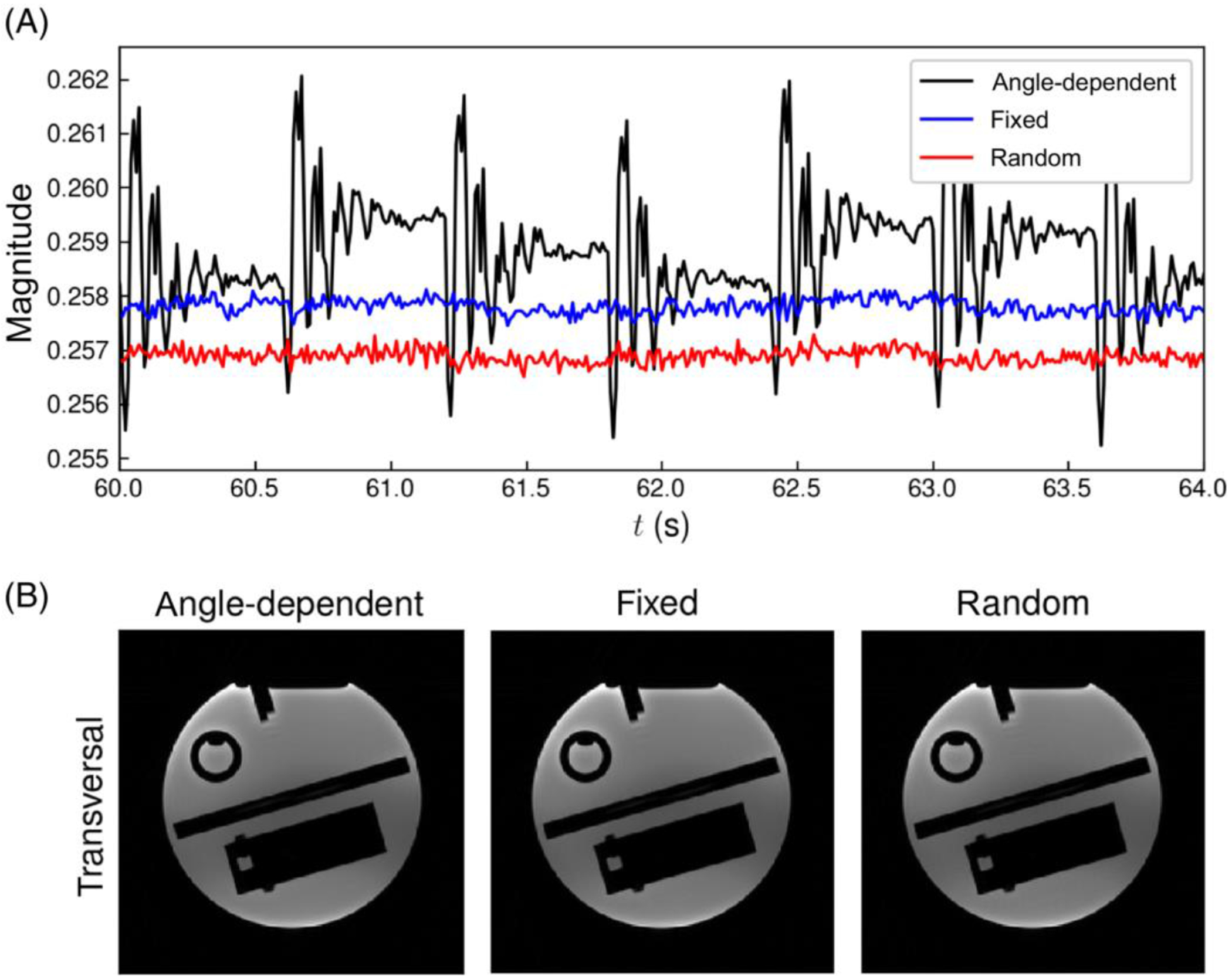
Free induction decay (FID) navigator signal magnitudes over time from three separate measurements of a stationary phantom with *T*_R_ = 10 ms with angle-dependent (black), fixed (blue), and random (red) spoiling schemes (A). Each data point represents the sum of the magnitude values of the 22 central samples of each FID readout from the same, single receiver coil. The different spoiling schemes did not lead to observable disparities in the resulting images (B).

Stationary phantom data showed that angle-dependent spoiling caused significant variations in the FID navigator signals, which were removed by using a fixed or random gradient spoiling scheme (Figure 4A). Unit-less variances were determined at (3.0 ± 1.6) × 10^−6^, (2.78 ± 1.6) × 10^−6^ and (2.4 ± 0.8) × 10^−5^ for fixed, random, and angle-dependent spoiler-gradient moments, respectively. Differences in mean FID-signal magnitudes between the three spoiling schemes were less than 1% (Figure 4A). Meanwhile, image quality of the 3D iNUFFTs did not visibly differ between the selected spoiling schemes (Figure 4B). Therefore, fixed gradient spoiling was selected for further measurements.

### 3.2 | Motion phantom

**FIGURE 5:**
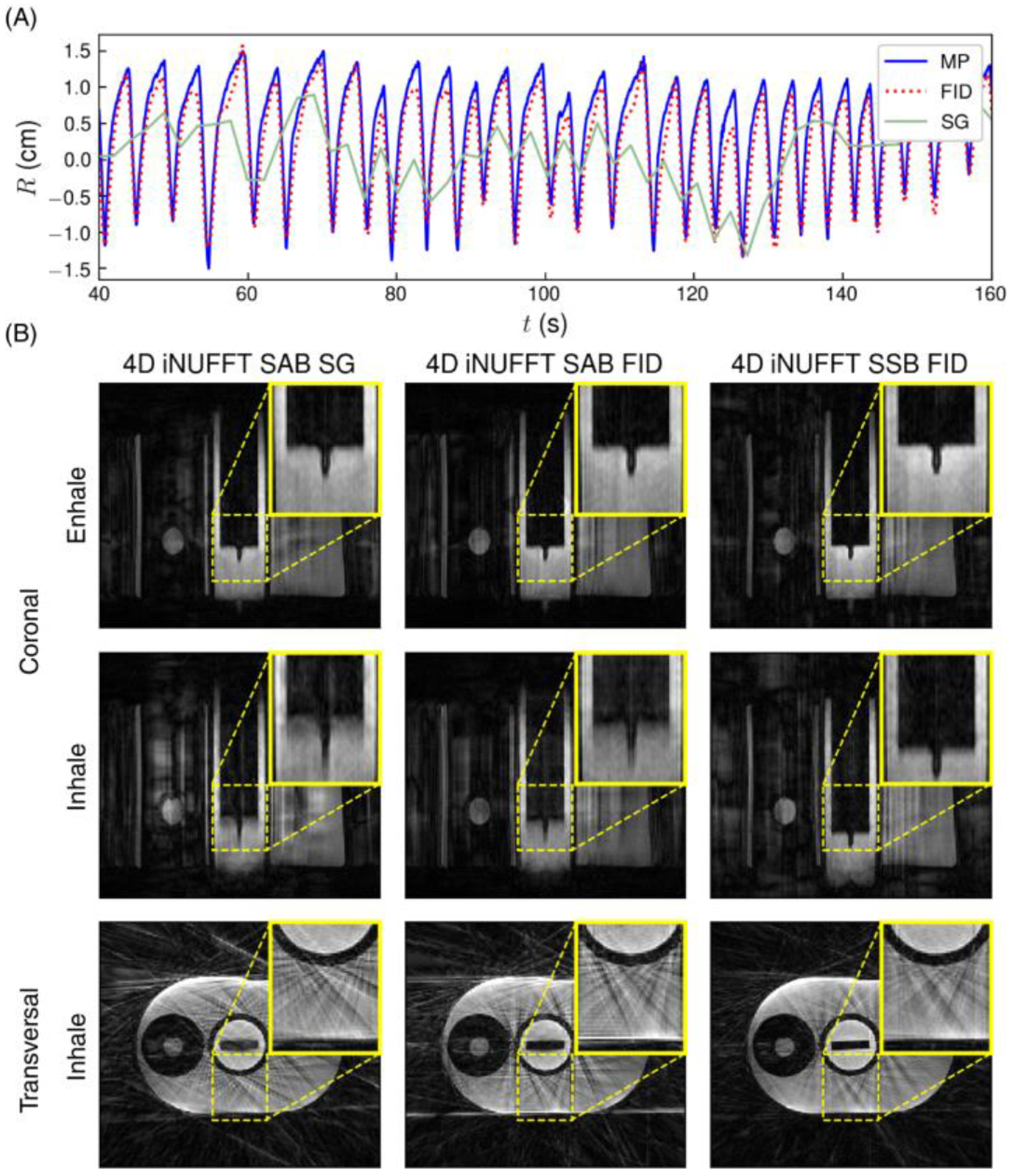
Respiratory signals (A) and nonuniform Fourier transform (iNUFFT) reconstructions (B) of the motion phantom data with both spoke-angle binning (SAB) and single-spoke binning (SSB). The blue, green, and dotted red lines in (a) represent the motion phantom’s reference (MP), self-gated (SG), and free induction decay (FID)-navigated respiratory signals, respectively. For coronal reconstructions, the vertical axes of images correspond to the slice encoding (*z*) direction. An animated version of (B) is available as Video S1.

Respiratory motion curves estimated from the FID navigator signals and the MR imaging data are shown in Figure 5A, along with the ground-truth motion pattern imposed on the phantom. The FID-navigated respiratory signal showed a high correspondence to the ground truth motion pattern, indicating that the rigid motion was accurately detected. Contrarily, the self-gated respiratory signal was strongly aliased. Correlation coefficients of the FID-navigated and self-gated respiratory signals with respect to the reference equaled 0.98 and 0.26, respectively.

The proposed SSB showed the sharpest edge delineation of the motion phantom’s oscillating cylinder when compared to SAB in 4D iNUFFT images (Figure 5B). In the transverse plane, SSB led to a reduction of streaking artifacts, likely because different partitions contained different sets of spoke angles, increasing incoherence. Self-gated SAB led to the largest loss in image sharpness out of the three binning types. Animations of the motion phantom’s 4D iNUFFTs are available as Video S1.

### 3.3 | In vivo

**FIGURE 6:**
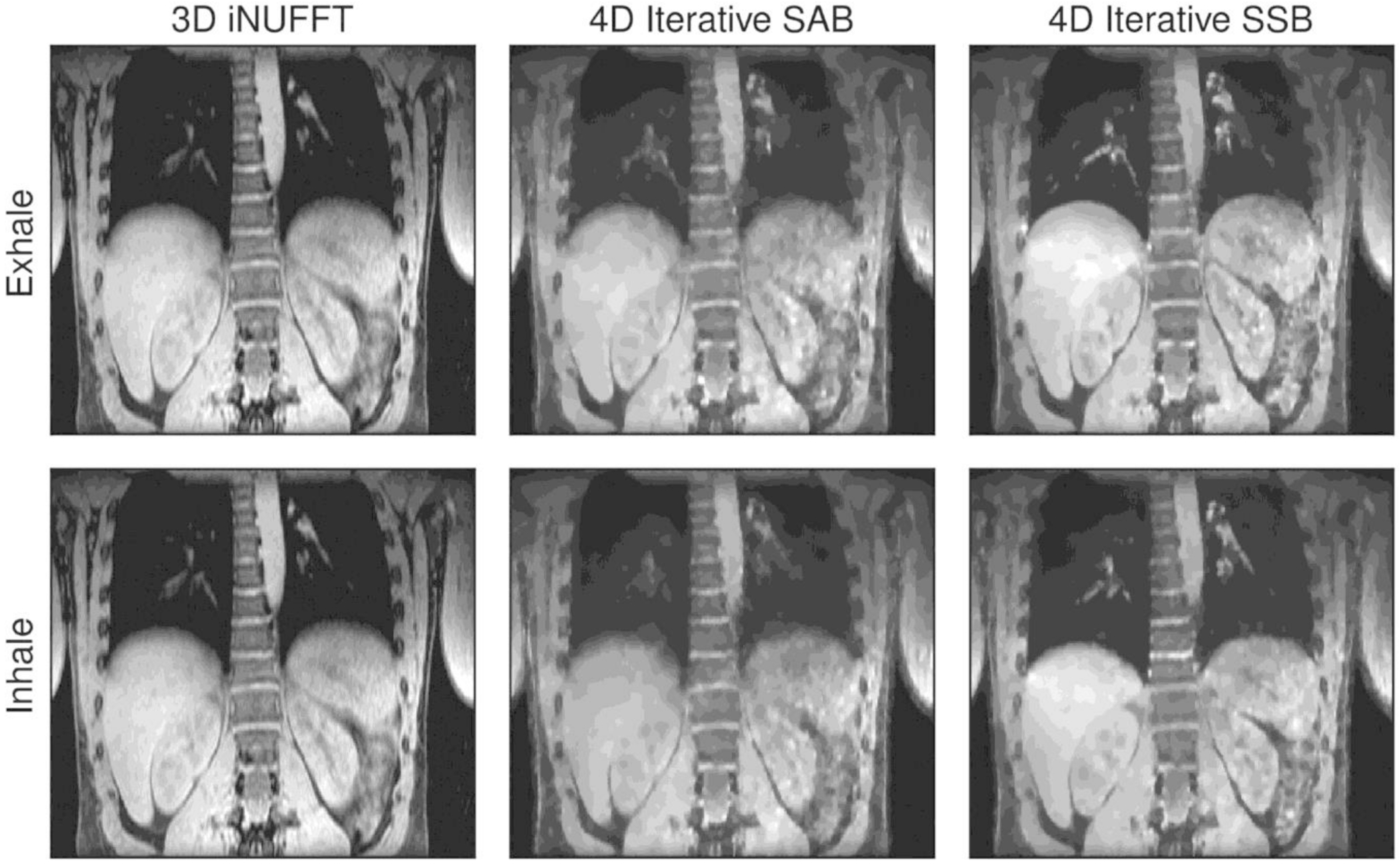
Coronal view of the iterative reconstructions from volunteer 1 for self-gated spoke-angle binning (SAB) and single-spoke binning (SSB). The top two rows display the in- and exhalation phases in coronal views with the vertical axis as the phase encoding (*z*) direction. The SSB reconstructions show considerably less motion artifacts than those of SAB. Animated reconstructions for this volunteer are available as Videos S2 and S3.

In in vivo measurements of free-breathing volunteers, 4D iterative SSB reconstructions showed an increased sharpness of the diaphragm and pulmonary vessels compared to 4D iterative self-gated SAB and motion-averaged iNUFFT (Figure 6). Self-gated SAB was able to reduce some of the motion blur in the end-exhalation phase, but reconstruction of the inhalation phase obscured small structures in the lungs. As expected, the iterative reconstruction algorithm led to a slight loss in sharpness of the 4D images due to regularization with the 𝓁_1_-norm of the spatial gradient.

**FIGURE 7:**
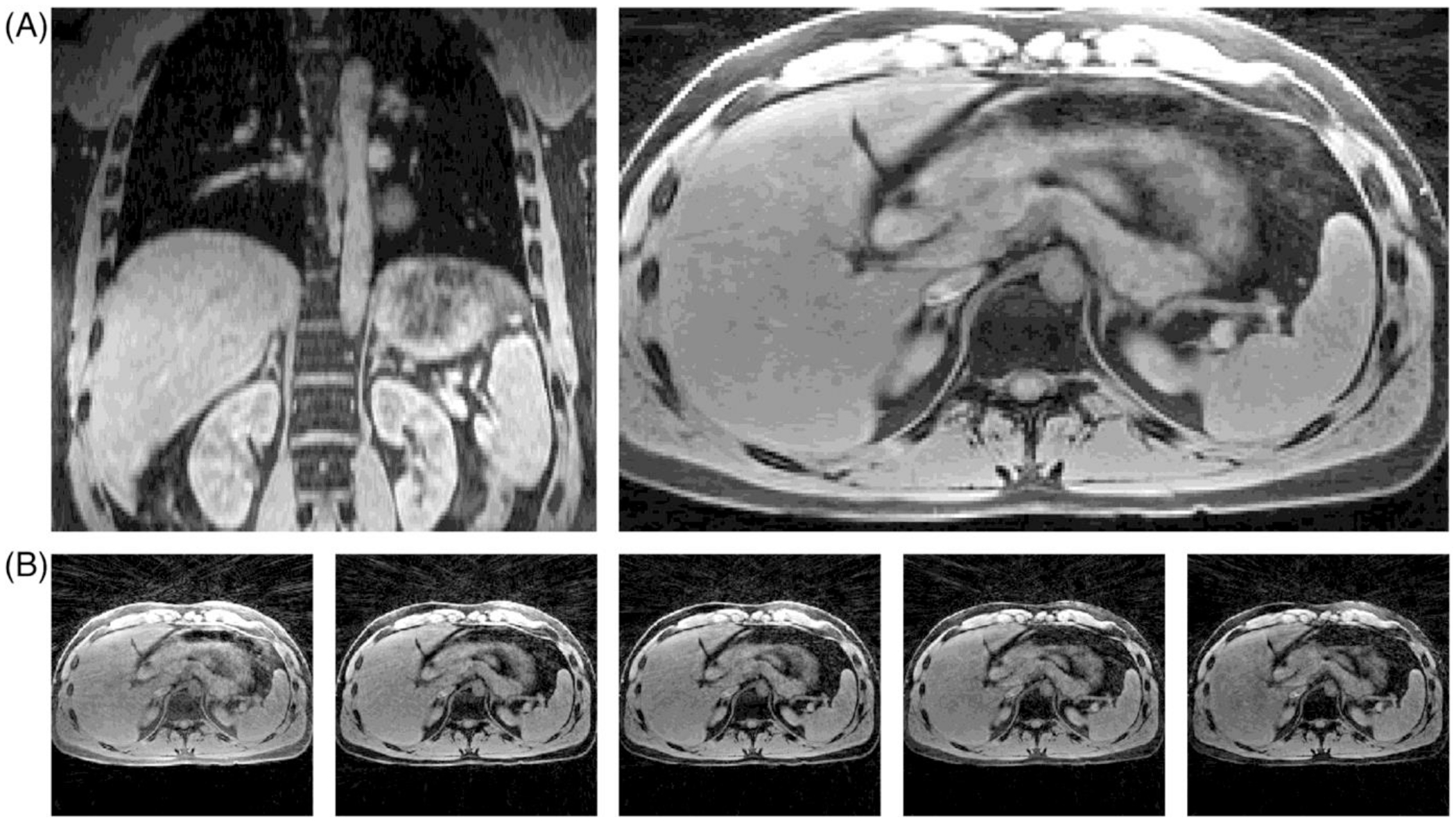
Root sum-of-squares-combined multi-echo single-spoke binning (SSB) inverse nonuniform Fourier transform (iNUFFT) reconstructions of the exhalation phase of volunteer 6 (A), and examples of the five individual echoes used to produce these images (B). The echo-combined motion-resolved iNUFFT images resulted in reduced aliasing artifacts compared to the single-echo images, enabling the use of the gradient entropy as a quantitative measure of image sharpness without having to use regularized reconstructions.

The echo-combined reconstructions of volunteer 6 show hardly any aliasing artifacts and are devoid of blur due to spatial regularization (Figure 7A). The angular undersampling was removed by means of the blip angle, which eliminated the need of the iterative reconstruction to resolve the aliasing. However, this came at the cost of mixing contrasts of different signal echoes. Any remaining blur within the images is due to partial volume effects and motion. Therefore, to estimate the level of motion-correction with SAB and SSB, the echo-combined reconstructions were selected for calculation of the gradient entropy in each volunteer.

**FIGURE 8:**
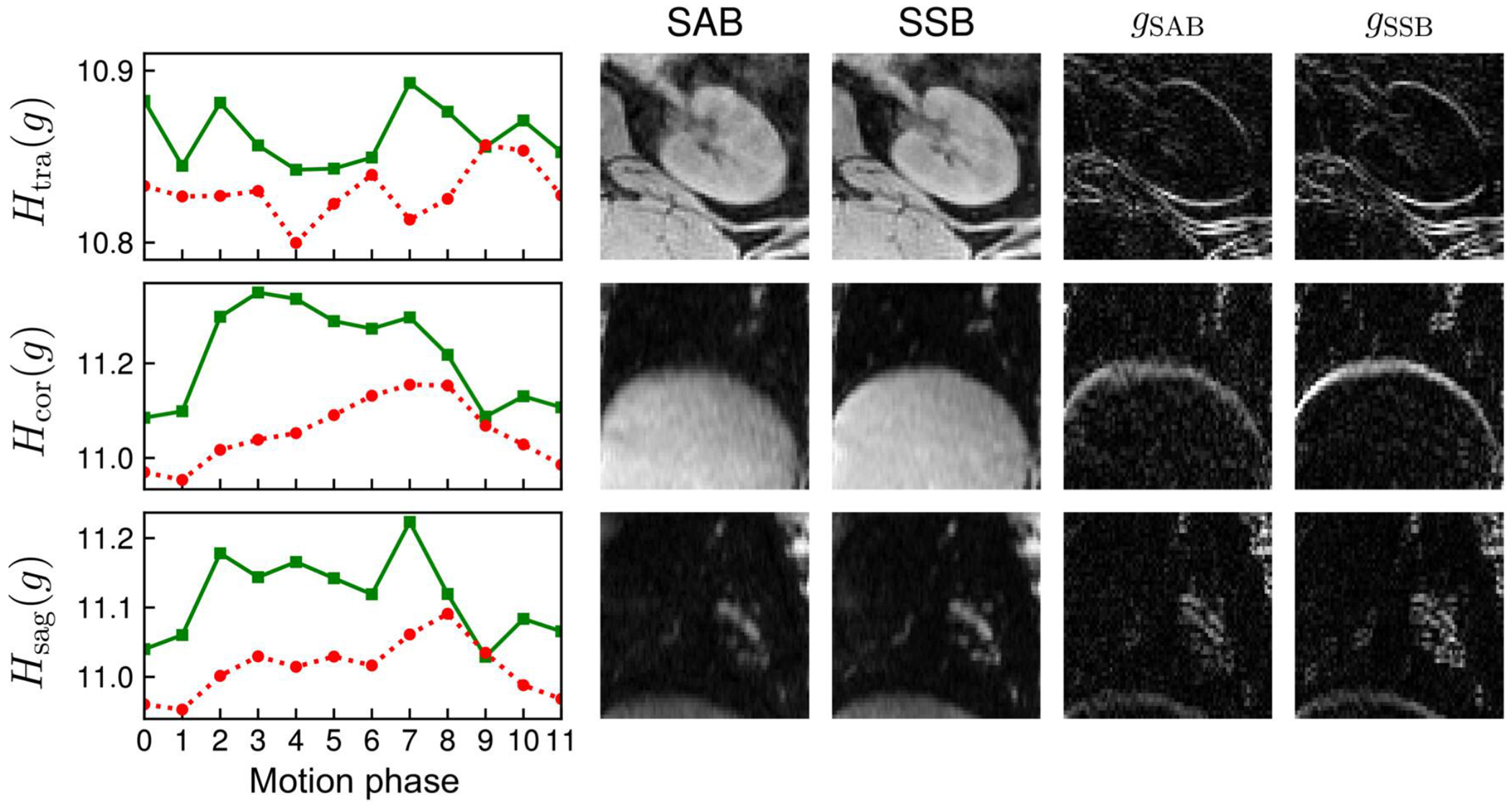
Gradient entropies for self-gated spoke-angle binning (SAB) (solid green line) and single-spoke binning (SSB) (dotted red line) across three ROIs in transversal (top row), coronal (middle row), and sagittal (bottom row) orientations of volunteer 2’s motion phase 4. The transversal, coronal, and sagittal ROIs are located at the left kidney, the liver dome, and the lung vessels, respectively. The columns labeled SAB and SSB are the gray-level images reconstructed with self-gated SAB and SSB, *g*_SAB_ and *g*_SSB_ represent the corresponding image gradients.

**FIGURE 9:**
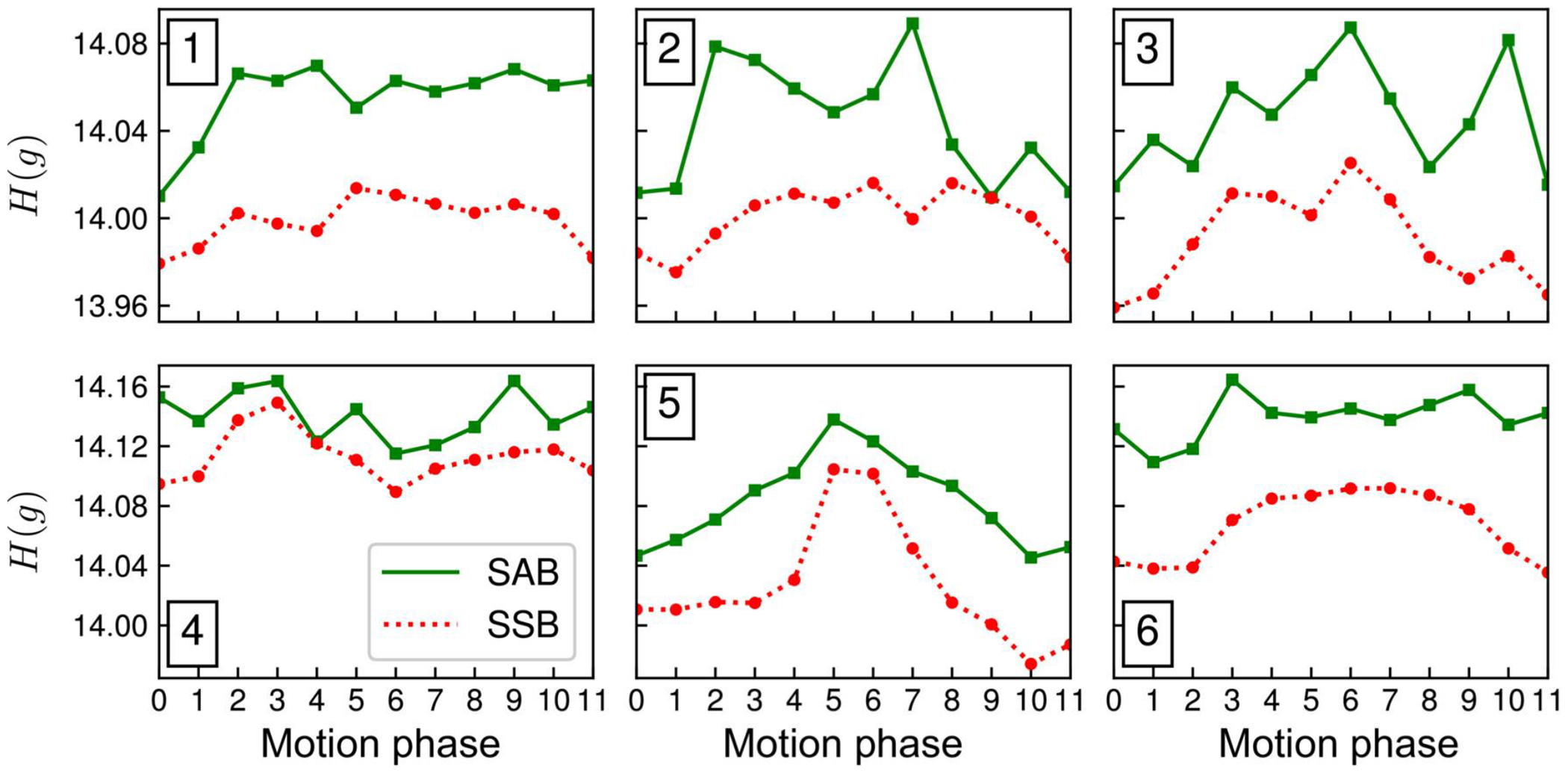
Gradient entropies *H*(*g*) for self-gated spoke-angle binning (SAB) (solid green line) and single-spoke binning (SSB) (dotted red line) for volunteers 1–6. Each data point represents the average value of *H*(*g*) across 70 central coronal slices. *H*(*g*) was lower for SSB than for SAB in all volunteers and motion phases, indicating an overall increase in image sharpness using SSB.

Overall image sharpness of echo-combined reconstructions increased across all three ROIs for volunteer 2, as indicated by the computed gradient entropy across each ROI (Figure 8). Similar trends in gradient entropy can be seen across the echo-combined reconstructions of all six volunteers (Figure 9): gradient entropies were lower for SSB than for self-gated SAB in all motion phases in all volunteers. Except for volunteer 4, the phases near exhalation showed the lowest gradient entropy or highest image sharpness. SSB achieved a statistically significant lower gradient entropy than SAB with p = 0.03, reflecting the overall increase in sharpness of the reconstructed images.

The FID navigators’ sampling frequency was sufficiently high to also estimate cardiac motion signals. To test the feasibility of cardiac gating using SSB, a bandpass filter was applied to the navigator data with the pass band set between 1.0 and 1.7 Hz. Cardiac signals were then created in the same way as the navigated respiratory signals. However, harmonics of the spectral artifacts could not be automatically removed from the selected frequency range. When creating respiratory signals, these artifacts were canceled out by the low-pass filter. To prevent these high-frequency modulations from becoming the first principal component in the cardiac analysis, a set of receivers with minimal artifacts was manually selected. Animations of both the respiratory- and cardiac-gated images for volunteer 1 are available as Videos S2 and S3.

Finally, an additional experiment was performed in a second motion phantom with pulse sequence parameters identical to the in vivo measurement described above, with motion phantom settings close to physiological breathing frequency and motion amplitude of the volunteers (Appendix S1).

## 4 | DISCUSSION

This work presents a novel method of motion-corrected imaging using a golden angle radial stack-of-stars GRE sequence. Instead of binning full spoke stacks, individual readouts are assigned to a motion phase, according to a motion signal obtained from FID navigator data acquired at each *T*_R_. In a 4D phantom and in healthy volunteers, SSB is shown to increase sharpness in motion-corrected reconstructions compared to the standard SAB. As a primary strength, SSB renders the motion binning accuracy essentially independent from the duration of *k_z_*-traversal, that is, from *T*_R_ and the number of partitions. SSB thus enables use of motion-corrected radial stack-of-stars MRI in a much broader domain of applications. For instance, it could be applied as a starting point for high resolution motion-corrected static imaging, for example, for liver T*_2_ mapping. Similarly, SSB could be utilized in high-resolution 4D cine imaging in abdomen or thorax for the assessment of organ motion, for example, for MR-guided radiation therapy planning.

Limitations in the slice resolution and volumetric coverage of motion-corrected radial stack-of-stars imaging have been addressed previously. First, in *k_z_*-accelerated variable-density stack-of-stars sampling, acceleration along the *k_z_*-dimension decreases acquisition time of each spoke stack,^23^ but still requires each stack to be binned into the same motion phase. It therefore does not provide an equal level of binning accuracy, nor the same flexibility in the choice of sequence parameters, as SSB does. Second, in MRI multitasking,^11,36^ an elegant method primarily used to quantify T_1_ relaxation times in heart and liver, a navigator readout line along *k_z_* was introduced in the golden-angle radial stack-of-stars sequence. The frequency of these navigators can be varied, for example one in nine readouts being a navigator.^11^ Since the magnetization’s steady-state needs to be maintained, the interleaved navigator readouts represent a trade-off between temporal resolution and scanning efficiency. For longer *T*_R_, a relatively large amount of scan time is spent on acquiring navigators instead of image data. In our proposed method, FID navigator data allows SSB at any *T*_R_ with a fixed minor increase in acquisition time.

SSB was applied using navigator data obtained from FID readouts integrated within the sequence. However, SSB can in principle be performed using any motion signal with sufficient updating frequency, and other methods may have several advantages over FID navigators. While the FID navigators provided robust detection of respiratory motion by virtue of an adapted gradient spoiling scheme, residual artifacts in the navigator signals remained at frequencies >1 Hz. These residual artifacts were likely related to the golden-angle increments and prevented accurate detection of cardiac motion. For robust cardiac-gated SSB, the unlocalized data should likely be replaced by a different navigator with a similar temporal resolution, e.g. a pilot tone.^37^ Replacing the FID readouts by the pilot tone would also remove the time loss associated with the FID navigators and the fixed gradient spoiling scheme.

Image sharpness of the in vivo reconstructions using SSB will further increase with a larger number of motion phases. The motion blur that remained in these images was most likely due to the finite temporal width of the binning window. In this work, the number of motion phases for the in vivo data was limited to avoid high undersampling factors and maintain sufficient SNR. To accommodate more motion phases, image registration could be included in the reconstruction process to further rectify aliasing and increase the SNR.^28^

One limitation of the proposed SSB is the prolonged repetition time due to the combined water excitation, FID sampling, and fixed spoiler gradients. Sampling of the navigator data and fixed gradient spoiling account for an additional 2.0 ms per *T*_R_, which could be eliminated by using an external navigation device instead of FID sampling. The 800 μs time loss due to the use of water-excitation pulse might be mitigated with magnetization-prepared fat saturation techniques. These alternatives have not been extensively explored within our work. For a quick fat saturation, the partition order should be carefully reconsidered. The pulse sequence should likely use a form of centric reordering, while at the same providing a sufficient level of incoherence between the sample order of partitions and the respiratory cycle. Alternatively, traditional excitation followed by Dixon-based water and fat reconstruction may be considered,^20,38,39^ at the cost of increased *T*_R_ and reconstruction complexity.

A second limitation of SSB is that the reconstruction problem is no longer separable along the slice dimension. A common way to reduce the memory requirement of radial stack-of-stars reconstructions is to perform an initial inverse Cartesian Fast Fourier Transform along the slice direction, after which the inverse problem can be solved independently for each slice.^6,19^ For SSB, however, the acquired spoke angles vary between partitions in each motion phase, precluding a straightforward inverse Cartesian Fast Fourier Transform along the slice axis.

## 5 | CONCLUSION

We described a novel approach for acquisition and reconstruction of radial stack-of-stars data with improved temporal resolution of respiratory motion detection and higher gating accuracy, based on integrated FID navigators and SSB. Evaluation in six volunteers demonstrated a consistent increase in image sharpness as quantitatively measured according to the gradient-entropy criterion. With this approach we eliminate trade-offs between spatial resolution, volumetric coverage and choice of contrast in motion-corrected GRE radial stack-of-stars MRI.

## Supplementary Material

Video 1 **Video S1.** Animations of the motion phantom’s 4D iNUFFT reconstructions using either self-gated SAB, FID-navigated SAB, or SSB. The images were reconstructed from factor-10 undersampled data and display the differences in aliasing artifacts between the three methods. SSB most clearly resolved each respiratory phase and shows an overall reduction of in-plane streak artifacts.

Video 2 **Video S2.** Animations of respiratory-gated, echo-combined reconstructions of volunteer 1. For each reconstruction, the data was binned into 12 motion phases with an undersampling factor of approximately 3.5. SSB resulted in the least amount of motion blur at the liver dome, and maintained visibility of the lung vessels in every motion phase. Compared to SSB, SAB showed increased motion blur.

Video 3 **Figure S3.** Animations of cardiac-gated, echo-combined reconstructions of volunteer 1. For each reconstruction, the data was binned into 12 motion phases with an undersampling factor of approximately 3.5. In contrast to SAB, SSB successfully resolved 12 cardiac phases.

Supp 1

## Figures and Tables

**TABLE 1:** Sequence parameters of the MR acquisitions of phantom and volunteer data

Parameter	Stationary phantom	Motion phantom	In vivo
Base resolution	256	256	256
Number of spoke angles	400	400	700
Acquired partitions	60	112	56–80
Number of echoes	1	1	5
Number of receivers	20	30	20–30
TE (ms)	5.0	2.46	2.12, 3.72, 5.32, 6.92, 8.52
TR (ms)	10.0	20.0	11.50
Δ*t* (ms)	600	2240	690–920
Flip angle (°)	16	15	7
Voxel size (mm^3^)	1.17 × 1.17 × 1.50	1.56 × 1.56 × 1.25	1.5 × 1.5 × 2.5
FOV (mm^2^)	300 × 300	400 × 400	384 × 384
Slice oversampling (%)	0	0	6.7–12.5
Slice resolution (%)	50	50	50
Bandwidth (Hz/px)	781	698	781
Orientation	Transversal	Transversal	Transversal
Acquisition time (min:s)	4:00	14:58	8:03 – 10:44

*Note*: Δ*t* indicates the dwell time between data points of the self-gated motion signal, that is, the time between subsequent spoke angles. Abbreviations: FOV, field of view; TE, echo time; TR, pulse repetition time.
