# Supplementary material for: Single-spoke binning: Reducing motion artifacts in abdominal radial stack-of-stars imaging
Source: Magn Reson Med. Author manuscript; Available in PMC 2025 Mar 30. (PMC11955222; doi:10.1002/mrm.29576)
Supplement: Supp 1 [file NIHMS2064105-supplement-Supp_1.pdf]

S4. Supplementary scan of a second motion phantom

An additional MR exam of a motion phantom was performed with pulse sequence parameters equal to the in vivo examinations and motion settings close to physiological breathing motion of the volunteers. The motion phantom contained a central oscillating cylinder with an air-water boundary. Motion was programmed to describe a respiratory-like pattern with 12 cycles per minute and a maximum peak-to-peak cylinder translation of 2 cm. Imaging parameters were: TE/TR = 5.0/16.0 ms, base resolution = 256, number of spoke angles = 700, acquired partitions = 60, number of echoes = 1, Δt = 960 ms, flip angle = 10°, voxel size = 1.56 x 1.56 x 2.5 mm^3, FOV = 384 x 384 mm^2, slice oversampling = 7.1%, slice resolution = 50%, bandwidth = 781 Hz/px, acquisition time = 11 min 12 sec. Data were acquired in transversal orientation. Image reconstructions were performed with inverse non-uniform Fourier transforms with 12 overlapping motion phases. Therefore, each phase contained 116 spokes, leading to an undersampling factor of approximately 3.5 per phase.

The self-gated and FID-navigated respiratory signals both roughly corresponded to the motion phantom's reference (Figure S2a), although the self-gated respiratory signals displayed a lack of accuracy in detecting rapid changes in the respiratory motion due to the increased time interval between successive samples. This is reflected in correlation coefficients of 0.80 and 0.87 for the self-gated and FID-navigated respiratory signals, respectively. Image quality of the inhale phase appeared highest in the FID-navigated SSB reconstructions (Figure S2b). However, with these sequence parameters and motion settings differences in image quality are less pronounced than in the first motion phantom scan.

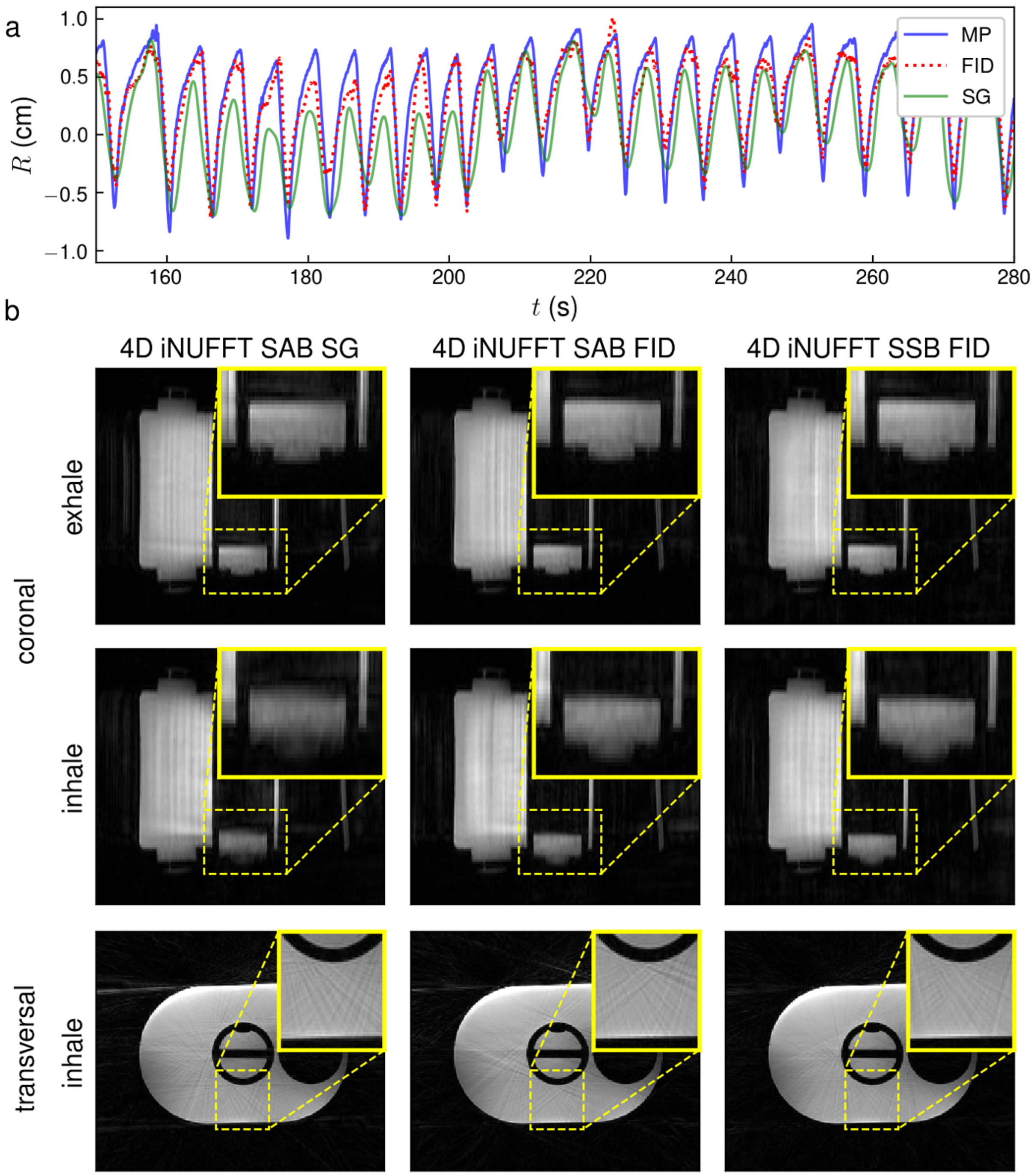

Figure S4. Respiratory signals (a) and inverse non-uniform Fourier transforms (b) of motion phantom data with both SAB and SSB. The blue, green, and dotted red lines in (a) represent the motion phantom's reference (MP), self-gated (SG), and FID-navigated (FID) respiratory signals, respectively.
